# Isolation of a novel thermostable dehydrochlorinase (LinA) from a soil metagenome

**DOI:** 10.1007/s13205-011-0012-x

**Published:** 2011-06-18

**Authors:** Ankit S. Macwan, Saleem Javed, Ashwani Kumar

**Affiliations:** 1 Environmental Biotechnology Section, CSIR-Indian Institute of Toxicology Research, Mahatma Gandhi Marg, Lucknow, 226001 India; 2 Department of Biochemistry, Hamdard University, New Delhi, 110062 India

**Keywords:** Hexachlorocyclohexane, Thermostability, Dehydrochlorinase, LinA, Metagenome

## Abstract

Hexachlorocyclohexane dehydrochlorinase (LinA) mediates first step of aerobic degradation of a chlorinated insecticide γ-hexachlorocyclohexane (γ-HCH). In this study, we describe characterization of a novel variant (LinA-type2) that is distinct from reported LinAs and is substantially more thermostable than archetypal LinA-UT26. LinA-type2 remains active even after 8 h of incubation at 45 °C, when nearly 50% activity of LinA-UT26 is lost after incubation for 60 min at the same temperature. Circular dichroism analysis revealed that secondary structures of LinA-UT26 and LinA-type2 are similar, but their *Tm* was 45 and 65 °C, respectively. Thermostability of LinA-type2 makes it suitable for bioreactors where allowance for higher temperatures can be of advantage.

## Introduction

**Fig. 1:**
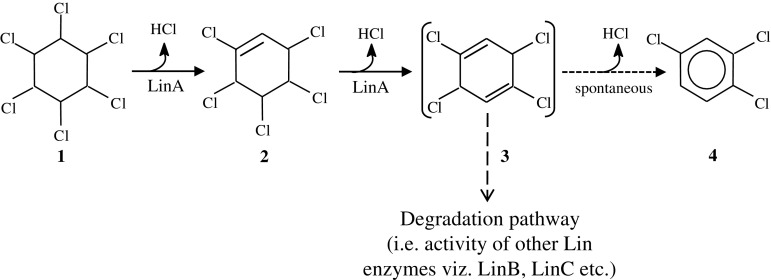
Reaction of HCH-dehydrochlorinase (LinA) with γ-HCH (formation of compounds 2, 3 and 4), and pathway followed in HCH-degradative bacteria. Compounds: **1** γ-Hexachlorocyclohexane (γ-HCH), **2** pentachlorocyclohexene (γ-PCCH), **3** 1,3,4,6-tetrachloro-1,4-cyclohexadiene (1,4-TCDN) and **4** 1,2,4-trichlorobenzene (1,2,4-TCB)

Chlorinated insecticide technical-hexachlorocyclohexane (t-HCH) consists of four major isomers; α- (60–70%), β- (5–12%), γ- (10–15%) and δ-HCH (6–10%), which differ in spatial distribution of chlorine atoms on cyclohexane ring (Willett et al. 1998). Sites contaminated by these isomers are present all around the world and are potential sources of toxicity (Willett et al. 1998; Lal et al. 2010). Several HCH-degrading microorganisms have been characterized from different parts of world (Lal et al. 2010). Pathway for the degradation γ-HCH has been worked out in considerable detail, and some information is also available about the degradation of other isomers. Briefly, enzyme ‘HCH-dehydrochlorinase LinA’ mediates first step in the biodegradative pathway of α-, γ- and δ-HCH i.e., their dehydrochlorination to 1,3,4,6-tetrachloro-1,4-cyclohexadiene via the corresponding pentachlorocyclohexene (Fig. 1). The formed product is further metabolized by sequential activity of other Lin enzymes of the pathway into readily utilizable products (Imai et al. 1991; Nagata et al. 2007; Lal et al. 2010). When LinA activity is tested in isolation, the formed 1,3,4,6-tetrachloro-1,4-cyclohexadiene is converted non enzymatically to 1,2,4-trichlorobenzene (Imai et al. 1991).

**Fig. 2:**
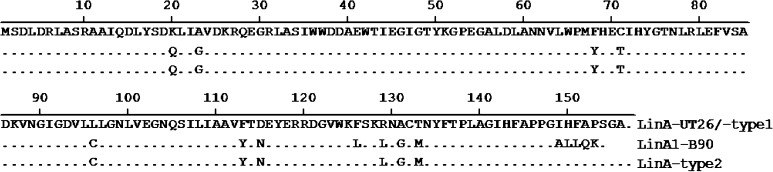
Amino acid sequence of LinA-UT26, LinA1-B90 and LinA-type2. Residues of LinA-UT26 and those differ in LinA1-B90 and LinA-type2 are shown

The archetypal LinA-UT26 was first reported from a strain *Sphingobium japonicum* UT26 (Imai et al. 1991), consists of 156 amino acids (Fig. 2), and its crystal structure has recently been described (Okai et al. 2010). Its activity is optimal at 35 °C and requires no additional cofactors for the activity. Several variant forms that are >85% identical to it have been described from various HCH-degrading organisms (Lal et al. 2010). LinA has potential for bioremediation of HCH-contaminated habitats, and its variants with improved properties are therefore desired (Mencia et al. 2006). Here, we report characterization of a thermostable LinA whose gene was obtained from metagenome of a HCH-contaminated site.

## Materials and methods

### Isolation of metagenomic DNA, and PCR amplification of *linA*

Soil samples, contaminated with HCH-isomers, were collected from the vicinity of a HCH-manufacturing unit near Lucknow, India. Metagenomic DNA was isolated from it by using ‘Fast DNA Spin Kit for soil’ (Qbiogene, Heidelberg, Germany). Amplification of *linA* was done by using primer F1 (1–25 bp from start codon of *linΑ*-UT26) and R1 (99–120 bp downstream from the stop codon of *linΑ*-UT26; Table 1). Temperature program for the amplification reactions was, initial denaturation at 95 °C for 5 min followed by 30 cycles of denaturation at 95 °C for 30 s, annealing at 55 °C for 30 s and extension at 72 °C for 3 min and a final extension at 72 °C for 10 min. Enzyme used for amplification was Pfu thermostable DNA Polymerase (Fermentas, Hanover, MD, USA) with 3′–5′ proofreading activity.

**Table 1:** Primers used in this study

Primer	Sequence	Restriction site	Characteristics	Accession no.
F1	CATATGAGTGATCTAGACAGACTTGCAA	NdeI	1–25 bp of *linΑ*-*UT26*	D90355
R1	CCCGGCGTGACTCATCTCCAAT	Nil	99–120 bp downstream from the stop codon of *linΑ*-*UT26*	D90355
R3	CTCGAGTGCGCCGGACGGTGCGA	XhoI	448–468 bp of *linΑ*-*UT26* (does not have stop codon TAA)	D90355

### Molecular cloning and nucleotide sequencing

The amplified products were purified from an agarose gel by using ‘GenElute^TM^ Gel Extraction Kit’ (Sigma–Aldrich, St. Louis, MO, USA), and nucleotide ‘A’ was added at their 3′ end by ‘Single dA^TM^ tailing kit’ (Novagen, Darmstadt, Germany). The dΑ-tailed products were ligated with TΑ-cloning vector (pCR4-TOPO, TOPO TA Cloning^®^ Kit for sequencing, Invitrogen, Carlsbad, CA, USA). These were transformed into One Shot^®^ TOP10 Electrocomp^TM^*E. coli* by electroporation, using Gene Pulser Xcell^TM^ (Bio-Rad, Hercules, CA, USA). Both strands of inserts from the clones were sequenced on ABI PRISM-3100 (Applied Biosystems, Foster city, CA, USA) sequencer by using universal forward and reverse M13 primers. Sequences obtained were aligned by Clustal W algorithm (DNAStar Inc. WI, USA). Accession numbers for *linΑ*-type1, -type2, are EU863865, EU863871, respectively.

### Expression of LinA proteins

Genes of LinA-type1 and -type2 were expressed in *E. coli* for further characterization. Briefly, these were amplified again by using primer F1 and R3 (Table 1) and TA cloned, as above. Plasmids from the sequence verified clones were digested with NdeI and XhoI, ligated with pET-26b (+) vector (Novagen, Darmstadt, Germany) and cloned first in *E. coli* DH5α cells and subsequently in *E. coli* BL21 (DE3) cells. These upon expression enabled the formation of C-terminal his-tagged products. The clones for each *linA*, after reconfirming their sequence, were grown in LB medium till the optical density 0.6 was achieved, and then were induced by 0.1 mM IPTG for 4 h. The cells were harvested, washed and resuspended in 50 mM sodium phosphate buffer (pH 8.0), and lysed by sonication (Ultrasonic processor UP100H, Hielscher, Stuttgart, Germany). Contents were centrifuged at 20,000g for 30 min, and the expressed proteins present in the clear supernatant were purified by Ni–NTA Superflow (QIAGEN, Hilden, Germany) columns at 4 °C. Size exclusion chromatography was done on AKTA FPLC (GE Healthcare, Piscataway, NJ, USA), using Superdex^TM^ 200, 10/300 GL column and 50 mM potassium phosphate buffer (pH 8.0) as both pre-equilibration and run buffer. Protein estimation was done by ‘Bio-Rad Protein Assay Reagent’ (Bio-Rad, Hercules, CA, USA) using bovine serum albumin (Sigma, USA) as standard. The purified proteins were diluted to 1.0 mg ml^−1^ in 50 mM sodium phosphate buffer (pH 8.0) containing 15% glycerol, and stored at −20 °C in aliquots.

### HCH-dehydrochlorinase activity

Activity of LinAs were determined by following the disappearance of substrate, as described earlier (Wu et al. 2007). Briefly, the reaction medium (1 ml) contained 50 mM Tris HCl; pH 8.0, 170 μM HCH-isomer (stock solution 1 mg ml^−1^ in DMSO), 10% glycerol and 10 μg of LinΑ proteins for α-, and γ-HCH, but 100 μg for δ-HCH. Reaction vials in triplicates were set up for each time point, which were withdrawn after incubation at 30 °C for different time intervals. The reaction was stopped by acidification to pH < 2. Residual HCH, along with formed metabolites, was extracted with 1 ml *n*-hexane and analyzed by gas chromatography (Kumar et al. 2005). Optimal temperature for activity was determined by setting the reaction at different temperatures, using α-HCH as substrate.

### Thermostability of LinAs

For determination of thermostability of LinAs, the reaction medium (1 ml) that contained 50 mM Tris HCl; pH 8.0, 10% glycerol and 10 μg of either LinΑ was pre-incubated at 45 or 70 °C for different time intervals. Afterwards, the reaction was initiated by addition of 10 μg α-HCH. After 10 min incubation at same temperature, the reaction was terminated and analyzed as above. For measurement of thermo tolerance, LinAs were incubated at different temperatures for 1 h, followed with their cooling by incubation at 30 °C for 30 min. Thereafter, the enzyme activities were measured as described above, using α-HCH as substrate.

### Circular Dichroism measurements

Circular Dichroism measurements were made on Chirascan^TM^ Spectrometer (Applied Photophysics, Surrey, United Kingdom) that was calibrated with ammonium (+)-10-camphorsulfonate at 25 °C with cell of 1 mm path length. The values were obtained by using 10 μM protein in 50 mM potassium phosphate buffer (pH 8.0), and normalized by subtracting the baseline recorded for the buffer under similar conditions. For evaluating their temperature-induced melting, molar ellipticity (222 nm) was monitored at different temperatures that were increased at a constant rate of 1 °C min^−1^ to 90 °C.

## Results

### Isolation of *linAs* from the soil metagenome

Analysis of 100 *linA*s that were amplified from soil metagenome revealed presence of two major *linA* variants, *linA*-type1 and -type2, whose relative abundance was 11 and 18%, respectively. While sequence of LinA-type1 was 100% identical to *LinΑ*-UT26, LinA-type2 was novel and differed from LinA-type1 by ten amino acids (Fig. 2). It also differs by nine amino acids from another reported variant LinA1-B90 (Kumari et al. 2002). Since LinA-type1 and LinA-UT26 are identical, only the term LinA-type1 is used in this manuscript for brevity and also to highlight its comparison with LinA-type2. Besides these variants, several other *linA*s (accession numbers EU863846–EU863896) that are >98% identical to either of these, and whose relative abundance was 1–6%, were also present in the metagenomic DNA.

### HCH-dehydrochlorinase activity

LinA-type1, -type2 and LinA1-B90 exhibited dehydrochlorinase activity with α-, γ- and δ-HCH, but not β-HCH (Table 2), and formation of reported metabolites (Nagata et al. 2007) was observed by gas chromatography and mass spectrometry (data not shown). Their activities differed substantially for various isomers (Table 2). Thus, activity for γ- and δ-HCH was highest by LinΑ-type1 that was followed with -type2 and LinA1-B90. Activities towards α-HCH, however, were comparable by all three LinAs (Table 2).

**Table 2:** Dehydrochlorinase activity of various LinA proteins with different HCH-isomers. Activities were determined at 30 °C

HCH-isomer	^a^Dehydrochlorinase activity (nmol min^−1^ mg^−1^ protein)		
	-type 1	-type 2	A1-B90
α	640	625	620
β	ND^b^	ND	ND
γ	3460	380	310
δ	140	42	40

^a^Values given are mean of triplicates and standard deviation was less than 10%

^b^*ND* not detectable

### Thermostability of LinAs

**Fig. 3:**
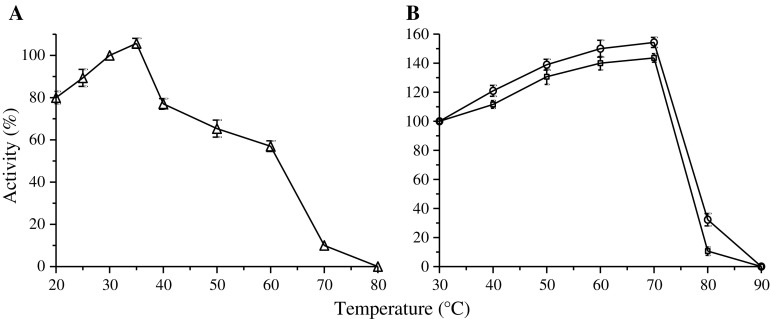
**a** Temperature optima of HCH-dehydrochlorinase activity of LinA-type1 (*triangle*), and **b** -type2 (*circle*) and LinA1-B90 (*square*). Activities at 30 °C (table 2) were taken as 100%

**Fig. 4:**
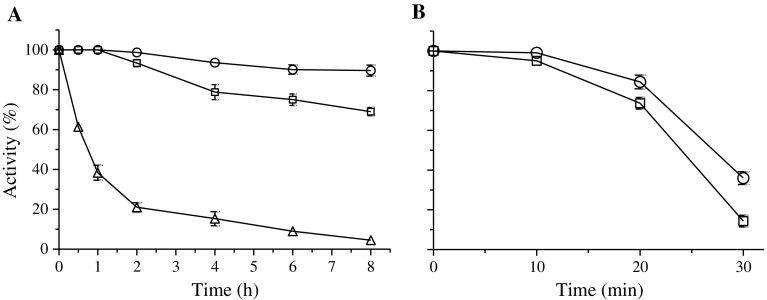
Thermostability of LinA proteins. Residual α-HCH dehydrochlorinase activity was measured after pre-incubation of LinA-type1 (*triangle*), -type2 (*circle*) and LinA1-B90 (*square*) at 45 °C (**a**) and of LinA-type2 and LinA1-B90 at 70 °C (**b**). Activities prior to pre-incubation (Table 2) were taken as 100%

Temperature optima for the activity of LinA-type1 and -type2 were 35 and 70 °C, respectively (Fig. 3). While no significant loss was observed in the activity of LinΑ-type2 after 8 h pre-incubation at 45 °C, >50% activity of LinΑ-type1 was lost after its incubation for 60 min at the same temperature (Fig. 4a). Further thermostability measurements revealed that after pre-incubation at 70 °C for different time periods, LinA-type2 is stable for 10 min but the activity declined gradually afterwards and only ~30% activity was observed after 30 min incubation (Fig. 4b). Similar decline in activity was also observed for LinA1-B90 at 70 °C.

**Fig. 5:**
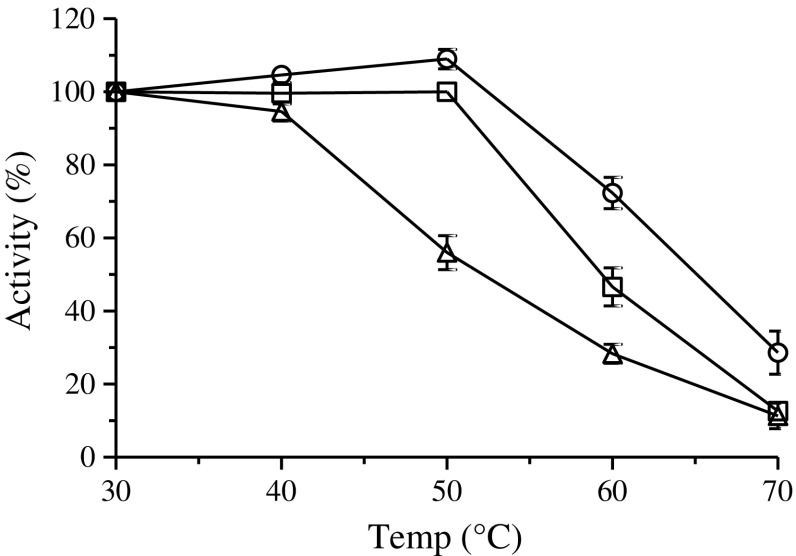
Thermotolerance of LinAs at different temperatures. LinA-type1 (*triangle*), -type2 (*circle*) and LinA1-B90 (*square*) were incubated for 1 h at different temperatures, air cooled by incubation at 30 °C for 30 min, and then α-HCH dehydrochlorinase activity was determined. Activities of different LinAs after pre-incubation at 30 °C were taken as 100%

Thermo tolerance of LinAs was evaluated by their pre-incubation at different temperatures for 1 h at 30–70 °C, followed by their cooling and recovery by incubation at 30 °C for 1/2 h. Under these conditions, 100% activity of all three LinAs was retained after pre-incubation at 30 and 40 °C (Fig. 5). At 50 °C, however, only LinA-type2 and LinA1-B90 retained full activity but 50% activity of LinA-type1 was lost. At 60 and 70 °C, loss of activity was observed for all three LinAs, which was highest for LinA-type1, followed by LinA1-B90 and LinA-type2, respectively.

**Fig. 6:**
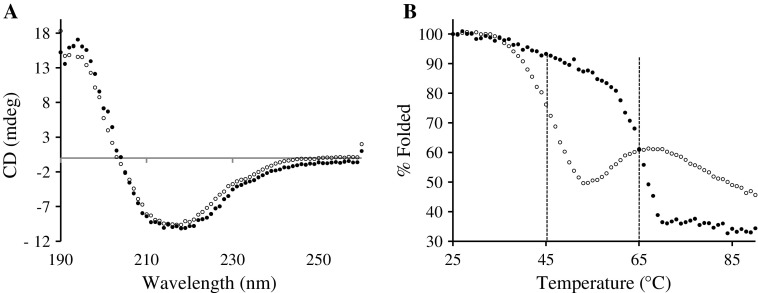
Circular dichroism spectroscopy plots of LinA-type1 (*open circle*) and -type2 (*closed circle*). **a** Far-UV spectra and **b** temperature-induced unfolding. *Vertical lines* in **b** represent temperatures where the proteins are half unwound

Circular dichroism spectra of LinA-type1 and -type2 were very similar (Fig. 6a), suggesting them to have similar secondary structures. Their thermal denaturation profiling (Fig. 6b), however, revealed that T_m_ for -type2 was substantially higher (65 °C), compared to that of -type1 (45 °C).

## Discussion

The study was designed to evaluate diversity of *linA* variants in metagenome of a t-HCH contaminated soil, and identify the ones with improved properties for the degradation of HCH-isomers. We describe characterization of a novel variant, LinA-type2, which is distinct from the reported LinAs and is highly thermostable. The thermostability was reflected in its (i) temperature optima at 70 °C, (ii) 100% retention of enzyme activity after 10 min incubation at 70 °C, and (iii) Tm of 65 °C by Circular Dichroism spectroscopy. LinA-type2 differs from archetypal LinA-UT26 by ten residues i.e., K20Q, A23G, F68Y, C71T, L96C, F113Y, D115 N, R129L, A131G and T133 M (Fig. 2), and any or more of these residues might be responsible for its higher thermostability. These ten changes, along with other residues e.g. ALLQK in place of IHFAP near its C-terminus, are present in LinA1-B90 (Fig. 2) and confer higher thermostability to it, which is being reported here for the first time.

Salt bridges have been identified as major contributory factor to thermostability of various proteins (Kumar et al. 2000). Crystal structure of LinA-UT26 revealed that the enzyme exists as a homotrimer, and each protomer forms a cone-shaped α + β barrel fold (Okai et al. 2010). Two inter-subunit salt bridges K26-D93′ and D19-R79′, where prime sign indicates a different subunit, have been identified in it (Okai et al. 2010). While these residues are conserved in LinA-type2, if any additional intra- or inter-subunit salt bridge(s) are formed, remains to be seen.

Ecological significance of the presence of several *linA* variants in soil metagenome is not immediately clear. Presence of LinA-type1 and -type2 are expected to be helpful in utilization of two α-HCH enantiomers, as described before (Suar et al. 2005). LinA genes have been suggested to be evolving rapidly (Nagata et al. 2007; Lal et al. 2010), and presence of other variants in the metagenome that are >98% identical to either of these might be reflecting this process. Higher thermal stability of LinΑ-type2, however, makes this enzyme suitable for use in bioreactors where allowance for higher temperatures is an advantage. The study paves way for designing better enzymes for improved degradation of HCH-isomers.
